# Use of Orange Oil Loaded Pectin Films as Antibacterial Material for Food Packaging

**DOI:** 10.3390/polym10101144

**Published:** 2018-10-14

**Authors:** Tanpong Chaiwarit, Warintorn Ruksiriwanich, Kittisak Jantanasakulwong, Pensak Jantrawut

**Affiliations:** 1 Department of Pharmaceutical Sciences, Faculty of Pharmacy, Chiang Mai University, Chiang Mai 50200, Thailand; Tanpong.c@gmail.com (T.C.); Yammy109@gmail.com (W.R.); 2 School of Agro-Industry, Faculty of Agro-Industry, Chiang Mai University, Chiang Mai 50100, Thailand; jantanasakulwong.k@gmail.com

**Keywords:** antibacterial activity, food packaging, orange oil, pectin film

## Abstract

This study aims to develop orange oil loaded in thin mango peel pectin films and evaluate their antibacterial activity against *Staphylococcus aureus.* The mango peel pectin was obtained from the extraction of ripe Nam Dokmai mango peel by the microwave-assisted method. The thin films were formulated using commercial low methoxy pectin (P) and mango pectin (M) at a ratio of 1:2 with and without glycerol as a plasticizer. Orange oil was loaded into the films at 3% *w/w*. The orange oil film containing P and M at ratio of 1:2 with 40% *w/w* of glycerol (P_1_M_2_GO) showed the highest percent elongation (12.93 ± 0.89%) and the lowest Young’s modulus values (35.24 ± 3.43 MPa). For limonene loading content, it was found that the amount of limonene after the film drying step was directly related to the final physical structure of the film. Among the various tested films, P_1_M_2_GO film had the lowest limonene loading content (59.25 ± 2.09%), which may be because of the presence of numerous micropores in the P_1_M_2_GO film’s matrix. The inhibitory effect against the growth of *S. aureus* was compared in normalized value of clear zone diameter using the normalization value of limonene content in each film. The P_1_M_2_GO film showed the highest inhibitory effect against *S. aureus* with the normalized clear zone of 11.75 mm but no statistically significant difference. This study indicated that the orange oil loaded in mango peel pectin film can be a valuable candidate as antibacterial material for food packaging.

## 1. Introduction

Currently, environmental problems and food safety have been of great public concern. Using packaging materials consisting of biopolymer is considered environmentally advantageous. Active packing has been defined as ‘a type of packaging in which the package, the product, and the environment interact to extend shelf life or improve safety and convenience or sensory properties, while maintaining the quality and freshness of the product [1]. One of the major concerns of the food industry is the spoilage of foods and food poisoning caused by microbial contamination, which occurs mainly on the surface as a result of the post processing and food handling process [2]. Thus, packaging possessing an antimicrobial property might be novel and gain interest for active packaging to be used in the food industry.

Pectin, one of the main structural water-soluble polysaccharides derived from plant cell walls, has been utilized in several fields, such as the pharmaceutic, cosmetic, and food industries, because of its excellent gelling property. Moreover, pectin can be extracted by simple methods from various plant materials. Mango peel, which is an agro-waste substantially arisen from fruit processing products in Thailand, was found to be a rich source of pectin [3,4,5].

Natural aromatic compounds and flavors such as fruit and vegetable essential oils are also extensively used in the food industry. For one clear example, orange oil exhibiting a significant bacterial inhibitory effect is one of the most beneficial and frequently used essential oils. Limonene, a major constituent found in orange oil, has been an active compound implicated in an antimicrobial property [6]. Limonene is a clear liquid at 25 °C with a citrus-like taste and odor. It is slightly soluble in glycerol, soluble in ethanol and carbon tetrachloride, and miscible with fixed oil. Limonene has demonstrated antibacterial activity as has been shown to inhibit the growth of many bacterial species in vitro, for example, *Staphylococcus aureus*, *Salmonella enteritidis*, *Escherichia coli*, *Klebsiella pneumoniae,* and *Proteus vulgaris* [7,8,9]. Limonene, a lipophilic compound, is able to penetrate through lipids of a bacterial cell, distribute cell structure, and render them more permeable. Then, it causes cell death by extensive leakage of intracellular fluid and ions [10]. There were some previous studies of film loading bioactive compounds that showed antimicrobial activity for preservative packaging. For example, gelatin film containing bergamot and lemongrass essential oils against *Escherichia coli*, *Listeria monocytogenes*, *Staphylococcus aureus*, and *Salmonella typhimurium.* Hydroxy methylcellulose film incorporated with kiam wood extract against *E. coli*, *S. aureus* and *L. monocytogenes* and starch film containing saponin against *E. coli*, *S. typhi*, and *E. erogenous* [11,12,13]. In the current study, the bio-based packaging materials have been developed by incorporating selected components, that is, essential oil derived from oranges, into a mango pectin film packaging model. The developed orange oil loaded pectin films were characterized and evaluated for their antibacterial activity against *S. aureus.*

## 2. Materials and Methods

### 2.1. Materials

Commercial low methoxy pectin (LMP; Unipectine OF300C; DE = 30% and DA = 0%) was purchased from Cargill^TM^ , Saint Germain, France. Orange oil was purchased from Thai-China flavors and Fragrance Industry Co., Ltd., Nonthaburi, Thailand. Calcium chloride (CaCl_2_) was purchased from Merck, Damstadt, Germany. Mueller Hinton agar (MHA) was purchased from Becton Dickinson, Holdrege, NE, USA. Tryptic soy broth (TSB) was obtained from HiMedia Laboratories Pty. Ltd., Mumbai, India. *S. aureus* (ATCC 25923) was obtained from the BIOTEC, Manassas, VA, USA. Distilled water served as the solvent for preparing film solutions. 

### 2.2. Mango Pectin

Mangoes (cv. Nam Dokmai) were collected from Chiang Mai province in Thailand, during June to August 2018. The mangoes were washed and the peels were stripped with a peeling knife, and then dried in a hot air oven at 50 ± 2 °C. The dried peel was ground to a fine powder using a high-speed food processor, and was then screened using a sieve (number 30; diameter 0.6 mm). Pectin from mango peel was extracted using the microwave-assisted method [14]. First of all, mango peel powder was mixed with acidified water pH 1.5. The mixture was extracted using microwave oven, irradiated at a frequency of 2450 MHZ at 500 W for 20 min. Then, it was centrifuged at 9000 rpm for 10 min. After that, supernatant was precipitated with ethanol and the precipitated pectin was washed by ethanol three times. The mango peel pectin was dried in hot air oven at 40 °C. The dried mango peel pectin was ground to a fine powder and kept in desiccator for further experiment. 

### 2.3. Identification of Major Compounds of Orange Oil

The major compounds of orange oil used in this study were analyzed by gas chromatography mass spectrometer (GC-MS analyzer) from Agilent-Technologies (Santa Clara, CA, USA). A split ratio of 1:650 was used to inject the sample of 1.0 μL. The compounds of the sample were separated using an HP-5 MS capillary column (30 m × 0.25 mm, film thickness 0.25 μm). The carrier gas was helium and the flow rate was set as 1.5 mL/min. The data obtained from GC-MS analysis were compared with the National Institute of Standards and Technology mass spectral library for compound identification.

### 2.4. Preparation of Films Loaded with Orange Oil

The minimum inhibitory concentration (MIC) of orange oil against *S. aureus* was evaluated before loading orange oil into the film formulations. The MIC was evaluated using the broth dilution method [8]. Orange oil was prepared in various concentrations from 0.5 to 150 mg/mL by dilution with Tween^®^ 80. The orange oil concentration above MIC was then selected and used for loading in the prepared film, using the ionotropic gelation with solvent casting techniques [15]. Briefly, film forming solution 3% *w*/*v* was prepared using commercial low methoxyl pectin (P) mixed with the extracted mango peel pectin (M) in various ratios with and without 40% glycerol (G), basic on dry pectin weight as the plasticizer. Orange oil (3% *w/w*) was added into pectin solution and then mixed until a homogeneous mixture was obtained. After that, the steps that were previously described were followed [15]. The film without mango pectin nor glycerol was used as a control film (P_3_M_0_O) in order to focus on the effect of mango pectin in thin film formulation. Furthermore, only mango pectin cannot form gel by this technique. Thus, the combinations of both pectin in the film formulation were prepared.

### 2.5. Characterization of Films Loaded with Orange Oil

The mechanical properties of the films loaded with orange oil were tested by a texture analyzer TX.TA plus (Stable Micro Systems, Surrey, UK). Each experiment was repeated six times. The tensile strength, percent elongation, and Young’s modulus was calculated [15]. The film morphology was examined using scanning electron microscopy (SEM, JSM-5410LV, JEOL Ltd., Peabody, MA, USA). The SEM were performed at 15 kV under low vacuum mode. The morphology of film’s matrix at magnifications of ×500 was evaluated. 

### 2.6. Limonene Loading Content

The limonene loading content in the films was determined using GC-MS, which was previously described [16]. The limonene loading was calculated by the following equation: %Limonene loading = the actual quantity of limonene/the theoretical quantity of limonene × 100.

### 2.7. Film Sterility Test

Films containing orange oil were cut into a circle with a diameter of 4.0 mm, which is the same size as the disc that was used in the anti-bacterial activity test. The cut films were sterilized by ethylene oxide and tested for sterility by the direct inoculation in culture medium method [17,18]. Tryptic soy broth media and its containing *S. aureus* were used as negative and positive control, respectively. Film samples were placed in the media and evaluated the microbial growth sign by visual observation after incubation at 37 ± 2 °C for seven days.

### 2.8. Anti-Bacterial Activity of Films Loaded with Orange Oil

In vitro anti-bacterial activity of films was determined using the agar disc diffusion method [19]. *S. aureus* was activated in TSB and incubated for 6–10 h. Absorbance was then measured at 600 nm. The activated *S. aureus* turbidity had to equal 10^8^ cfu/mL and was used and cultivated on MHA plates. The films loaded with orange oil were put onto the MHA plate. Ampicillin was also used as a positive control. After that, the MHA plates were incubated at 37 °C for 24 to 48 h, and the diameter of the clear zone was measured, including the diameter of the film or disc, using a Vernier caliper. Each experiment was performed in triplicate.

### 2.9. Statistical Analysis

An analysis of variance (ANOVA) was carried out with SPSS software version 16.0 (SPSS Inc., Chicago, IL, USA).

## 3. Results and Discussion

### 3.1. Identification of Orange Oil

GC-MS analysis of orange oil showed 19 known compounds including hydrocarbons, aldehydes, alcohol, and ketone (Table 1). The major compound of the oil was limonene (84.57%), which was exhibited at the retention time of 4.801 min. Other compounds were also found in the oil, such as *cis*-limonene oxide (1.86%), β-myrcene (1.06%) and *trans*-limonene oxide (0.88%). Thus, limonene was used as the marker for further experiments in this study. Previous studies have also identified limonene to be the major component (67.44–94.50%) in orange oil [20,21,22].

### 3.2. Morphology and Mechanical Properties of Film Loaded with Orange Oil

**Figure 1:**
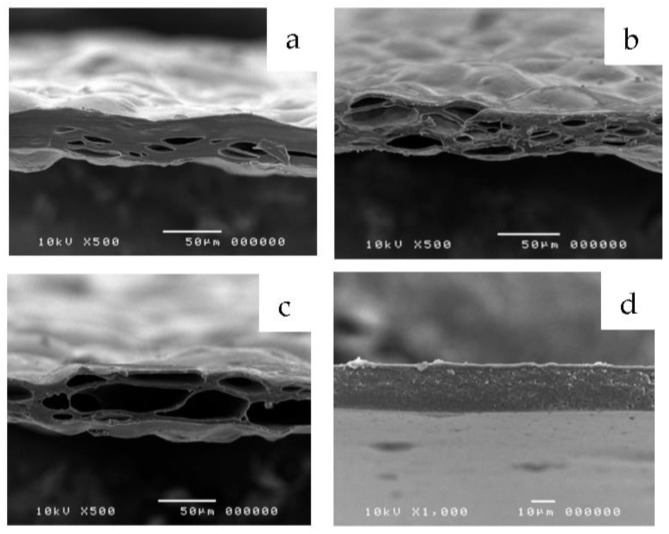
Scanning electron microscopy (SEM) of film’s matrix of P_3_M_0_O (**a**), P_1_M_2_O (**b**), P_1_M_2_GO (**c**), and P_1_M_2_ (**d**) films.

All film formulations’ compositions are shown in Table 2. The film without orange oil (P_1_M_2_ film) exhibited a smooth surface with dense matrix, whereas orange oil films showed rough surface with the presence of micropores inside the film’s matrix. When orange oil was loaded, we observed the increasing of the thickness of orange oil films, which was related to the size and amount of micropores inside the film’s matrix (Table 2 and Figure 1). The more orange oil evaporated, the greater the number and size of micropores, which resulted in a loose structure of the matrix and increased film thickness. Our results are consistent with Jouki et al. 2014, which found that quince seed film showed the loose film’s matrix when oregano oil was incorporated [23]. Furthermore, in a previous study, the effect of grape pomace extract in chitosan film was investigated. Film structure discontinuities were induced by incorporation of wax or oil into the polysaccharide matrix [24]. Moreover, this study found that the greater the mango peel pectin ratio, the greater the number of micropores as seen in P_3_M_0_O film dense film matrix with less micropores than P_1_M_2_O film. The mechanical properties of films loaded with orange oil are shown in Table 3. Young’s modulus of P_1_M_2_GO film significantly decreased to 35.24 MPa compared with 145.34 MPa for this film without orange oil. These results may be because of the amount of micropores in the polymer matrix. A previous study showed that quine seed mucilage films incorporated with the highest amount of oregano essential oil, which had many micropores, exhibited the best mechanical properties with the lowest tensile strength and Young’s modulus, as well as the highest percent elongation. The works of [23,25] also found that percent elongation of citrus pectin film incorporated with clove bud oil increased as a result of oils existing in the film matrix in the form of oil droplets, which can be easily deformed and improve the film’s extensibility.

### 3.3. Limonene Oil Loading Content

The limonene loading contents of films loaded with orange oil are shown in Table 3. The highest limonene loading content (86.17%) was obtained from P_3_M_0_O film, whereas P_1_M_2_GO film exhibited the lowest limonene loading content (59.25%). This result found that the texture of the orange oil film played an important role in limonene loading contents. Micropores indicated that orange oil had evaporated from the polymer matrix. Film with a lower amount and smaller size of micropores, like P_3_M_0_O, can encapsulate more limonene content. While the P_1_M_2_GO film with larger amount and size of micropores showed the lowest limonene content.

### 3.4. Film Sterility and Anti-Bacterial Activity

The product sterility test was used to confirm that the film samples were sterile, before testing the anti-bacterial activity. In sterility test, there was no microbial growth in any of the film samples. Growth of *S. aureus* was observed in the positive control, while no sign of *S. aureus* growth was observed in the negative control. The results of examination of the anti-microbial activity of films against *S. aureus* using the agar disk diffusion method are shown in Table 4. All films loaded with limonene exhibited a clear zone. In the direct comparisons of the clear zone diameter of film formulations containing different limonene loading content, the P_3_M_0_O film, which had the highest limonene loading content (86.17%), showed the widest clear zone diameter (10.02 mm), whereas P_1_M_2_GO film, which exhibited the lowest limonene loading content (59.25%), showed the smallest clear zone diameter (8.08 mm). Thus, the anti-bacterial activity of the films in this study was dependent on the amount of limonene remaining in the film. Generally, in a hydrophilic polymer such as pectin, water molecules from agar penetrate into the polymer matrix resulting in swelling of film; thus gradually widening the meshes of the polymer network and leading to greater release of active compounds into the surroundings [22]. However, in order to normalize limonene content in each film, the normalized clear zone was calculated and presented in Table 4. Interestingly, the normalized clear zone dimeter of P_1_M_2_GO film (11.75 mm) was higher than the others, but no statistically significant difference existed between the films. It may be concluded that when we consider film composition in the same amount of limonene loading content, the P_1_M_2_GO film, which showed better mechanical properties, tended to show higher anti-bacterial activity. In addition, our recent study found that loading orange oil in the form of microemulsion was able to reduce micropores in the film’s matrix and increase limonene loading capacity as well as anti-bacterial activity [16].

## 4. Conclusions

Films loaded with orange oil were prepared and their morphology, tensile properties, loading content, and anti-bacterial activity were investigated. The addition of orange oil decreased the Young’s modulus value, which appears to be related to the amount of micropores in the film’s matrix. Interestingly, the P_1_M_2_GO film, which had the best mechanical properties, exhibited the lowest limonene loading content (59.25%). For the test of anti-microbial against *S. aureus*, the P_1_M_2_GO film showed the highest normalized clear zone (11.75 mm), but no significant difference existed. This study indicated that the orange oil loaded in mango peel pectin film can be a valuable candidate as an antibacterial material for food packaging. However, this orange oil film needs further development, especially the increasing of the limonene loading content. Other technologies such as preparation of orange oil in micro-emulsion or nano-emulsion could be used in order to enhance the limonene loading content.

## Figures and Tables

**Table 1:** Identified compounds of orange oil. GS-MC—gas chromatography mass spectrometer.

Compound	GC-MS RT (min)	Peak Area (%)
2-Thujene	3.623	0.46
3-p-Menthene	3.708	0.06
β-Myrcene	3.874	1.06
Octanal	4.097	0.21
3-Carene	4.309	0.06
Limonene	4.801	84.57
1,2-Dimethylcyclobutane	5.597	0.13
Linaolool oxide	5.705	0.08
Linalool	6.375	0.77
*cis*-Limonene oxide	7.313	1.86
*trans*-Limonene oxide	7.433	0.88
α-Terpineol	8.990	0.25
Decanol	9.396	0.33
Carveol	9.842	0.67
Carvone	10.592	0.76
Farnesol	13.350	0.49
9,11-Dodecadien-1-ol	15.719	0.10
1,5-Cyclooctadiene, 1,5-dimethyl	16.125	0.06
Valencene	18.225	0.10

**Table 2:** Film composition and thickness. LMP—low methoxy pectin.

Film Code	Composition				Thickness (μm)
	LMP (% *w*/*v* of Pectin Solution)	Mango Peel Pectin (% *w*/*v* of Pectin Solution)	Glycerol (% *w/w* Based on Pectin Weight)	Orange Oil (% *w/w* of Pectin Solution)	
P_3_M_0_O	3	0	-	3	52.94 ± 0.21
P_1_M_2_O	1	2	-	3	58.82 ± 0.23
P_1_M_2_GO	1	2	40	3	70.58 ± 0.18
P_1_M_2_	1	2	-	-	25.71 ± 0.11

**Table 3:** Orange oil loading content, tensile strength, elongation, and Young’s modulus of films containing orange oil.

Film Code	Orange Oil Loading (%)	Tensile Strength (MPa)	Elongation (%)	Young’s Modulus (MPa)
P_3_M_0_O	86.17 ± 3.41 ^a^	6.12 ± 1.18 ^a^	2.28 ± 0.15 ^a^	266.04 ± 35.39 ^a^
P_1_M_2_O	70.10 ± 1.03 ^a^	3.12 ± 1.23 ^b^	2.57 ± 0.10 ^a^	126.91 ± 42.32 ^b^
P_1_M_2_GO	59.25 ± 2.09 ^b^	4.54 ± 0.18 ^c^	12.93 ± 0.89 ^b^	35.24 ± 3.43 ^c^
P_1_M_2_	-	4.98 ± 0.54 ^c^	6.15 ± 0.88 ^c^	145.34 ± 31.77 ^b^

Note: For each test, the different letters are statistically different (*p* < 0.05).

**Table 4:** Anti-bacterial activity of films against *S. aureus.*

Sample	Clear Zone Diameter (mm)	Normalized clear Zone (mm)
P_3_M_0_G film	ND	ND
P_3_M_0_O film	10.02 ± 0.03	10.02
P_1_M_2_O film	8.76 ± 0.01	10.77
P_1_M_2_GO film	8.08 ± 0.01	11.75
P_1_M_2_ film	ND	-
Orange oil (100 mg/disc)	9.64 ± 0.01	-
Tween^®^ 80	ND	-
Ampicillin (6.25 μg/disc)	9.82 ± 0.01	-

Note: ND = not detected. Normalized clear zone (nm) = (limonene content of P_3_M_0_O/limonene content of each film) × clear zone diameter of each film.
